# Non-destructive detection of protein content in mulberry leaves by using hyperspectral imaging

**DOI:** 10.3389/fpls.2023.1275004

**Published:** 2023-10-12

**Authors:** Xunlan Li, Fangfang Peng, Zhaoxin Wei, Guohui Han, Jianfei Liu

**Affiliations:** Research Institute of Pomology, Chongqing Academy of Agricultural Sciences, Chongqing, China

**Keywords:** hyperspectral imaging, mulberry leaf, protein content, non-destructive detection, visible and near-infrared

## Abstract

Protein content is one of the most important indicators for assessing the quality of mulberry leaves. This work is carried out for the rapid and non-destructive detection of protein content of mulberry leaves using hyperspectral imaging (HSI) (Specim FX10 and FX17, Spectral Imaging Ltd., Oulu, Finland). The spectral range of the HSI acquisition system and data processing methods (pretreatment, feature extraction, and modeling) is compared. Hyperspectral images of three spectral ranges in 400–1,000 nm (Spectral Range I), 900–1,700 nm (Spectral Range II), and 400–1,700 nm (Spectral Range III) were considered. With standard normal variate (SNV), Savitzky–Golay first-order derivation, and multiplicative scatter correction used to preprocess the spectral data, and successive projections algorithm (SPA), competitive adaptive reweighted sampling, and random frog used to extract the characteristic wavelengths, regression models are constructed by using partial least square and least squares-support vector machine (LS-SVM). The protein content distribution of mulberry leaves is visualized based on the best model. The results show that the best results are obtained with the application of the model constructed by combining SNV with SPA and LS-SVM, showing an *R* ^2^ of up to 0.93, an RMSE of just 0.71 g/100 g, and an RPD of up to 3.83 based on the HSI acquisition system of 900–1700 nm. The protein content distribution map of mulberry leaves shows that the protein of healthy mulberry leaves distributes evenly among the mesophyll, with less protein content in the vein of the leaves. The above results show that rapid, non-destructive, and high-precision detection of protein content of mulberry leaves can be achieved by applying the SWIR HSI acquisition system combined with the SNV-SPA-LS-SVM algorithm.

## 1 Introduction

Mulberry leaves are rich in a variety of bioactive ingredients necessary for the human body, with such functions as anti-obesity (Li et al., 2019), anti-oxidation and antibacterial (Thabti et al., 2014), and anti-diabetes (Riche et al., 2017), and thus are considered to be beneficial in the Asian population. Compared to animal protein, the abundant protein of mulberry leaves contains no animal cholesterol, with an amino acid pattern similar to that of the human body (Gryn-Rynko et al., 2016; Sun et al., 2017). In recent years, mulberry leaves are eaten as a vegetable, and used as a traditional source of animal feed protein as well in Asian countries (Srivastava et al., 2003; Yu et al., 2018). The protein content is one of the most important indicators for assessing the quality of mulberry leaves used as an animal feed source or a fresh vegetable.

At present, the methods for determining protein content in leaves are mainly chemical analysis methods (Ledoux and Lamy, 1986; Chromý et al., 2015; Denholm et al., 2021), such as the Kjeldahl nitrogen determination method. Such methods require the samples to undergo not only drying, grinding, and other destructive treatments, but also deboiling, distillation, and titration under the condition of concentrated sulfuric acid being added. This is a complex process producing chemical pollution. In light of this, it is highly necessary to introduce a non-destructive and rapid determination of protein content of mulberry leaves.

Hyperspectral imaging (HSI) combining imaging technology with spectral technology can provide both spectral and spatial information of substances. With the advantages of non-destructiveness, high efficiency, and low cost, HSI is widely used in non-destructive detection of protein content of different farm products, including meat products such as pork (*R* ^2^_P_ = 0.9161 and RMSEP = 2.71 mg/g) (Ma J. et al., 2019), lamb (R^2^_p_ = 0.67 and RMSEP = 0.41) (Pu et al., 2014), and beef (R^2^_P_ = 0.86 and SEP = 0.29) (ElMasry et al., 2013), and grain products such as wheat (*R* ^2^_P_ = 0.79 and RMSEP = 0.94) (Caporaso et al., 2018), rice (R^2^_P_ = 0.8011 and RMSEP = 0.52) (Ma et al., 2021), and peanuts (R^2^_P_ = 0.912 and RMSEP = 0.438) (Cheng et al., 2017). There are studies showing that N-H bonds in proteins present absorption peaks at 1,460–1,570 nm and 2,000–2,180 nm (Shenk et al., 2007; Chelladurai and Jayas, 2014), which lead to the non-destructive detection of proteins to be conducted by mainly using the Short-Wave Infrared (SWIR) HSI system with an acquisition wavelength range of 1,000–1,700 nm or 900–2,500 nm. There are also some other researchers using visible near-infrared (Vis-NIR) HSI with an acquisition wavelength range of 400–1,000 nm for non-destructive detection of proteins of meat (Ma J. et al., 2019), rice (Onoyama et al., 2018), milk (Jin et al., 2022), and rape leaves (Zhang et al., 2015), with good results obtained. As the main parts of optical imaging systems, detectors are meant for detecting and measuring the radiation reflected or transmitted by objects. A detector made of a certain material can only detect certain wavelength ranges, and the prices of detectors vary greatly. Currently, silicon detectors (300–1,100 nm) are the most widely used Vis-NIR detectors, and their prices are very low, compared with the slightly more expensive InGaAs detectors (900–2,500 nm) and the much more expensive HgCdTe detectors (1,000–2,600 nm). At present, there are only a few studies on the non-destructive detection of proteins of mulberry leaves. Ma et al. used a 900-1,600 nm handheld near-infrared spectrometer to detect proteins of dry mulberry leaves, and by combining with partial least squares (PLS) regression and the wavelength optimization method, they obtained a prediction set *R* ^2^ of up to 0.92 (Ma Y. et al., 2019). However, this method requires the mulberry leaves to undergo drying and grinding, and the obtained data are single point data, thus leading to failure to obtain the protein content of the whole leaves. The vibrational characteristics of different molecules and functional groups vary, resulting in differences in sensitivity to specific wavelengths among different substances. Therefore, we are not clear about the best detector material and the spectral range for conducting the non-destructive detection of protein content of mulberry leaves. As a result, it is necessary to choose an optimal compromise.

This study aims at developing a non-destructive and rapid method for the detection of protein content of mulberry leaves. The main research contents are as follows: (1) analyzing the spectral characteristics of mulberry leaves at Vis-NIR (400–1,000 nm) and SWIR (900–1,700 nm); (2) comparing different pretreatment, feature extraction, and modeling methods and selecting the best optimal data processes and methods; (3) selecting the best spectral range of HSI acquisition system for the detection of protein content of mulberry leaves; and (4) visualizing the distribution of protein content of mulberry leaves by using the optimal model.

## 2 Materials and methods

### 2.1 Materials

The healthy mulberry leaves, randomly collected and washed with tap water when brought to the laboratory, undergo hyperspectral images collection and protein content determination after the surfaces of the leaves become dry. In this study, 193 samples are randomly divided into the training set and the testing set at the ratio of 7:3, with 135 and 58 samples, respectively. Among them, the training set is used for training the model, with the 10-fold cross-validation method applied to the training set to adjust the model parameters and select the optimal model, while the test set is used for assessing the final model.

### 2.2 Acquisition and calibration of hyperspectral images

**Figure 1:**
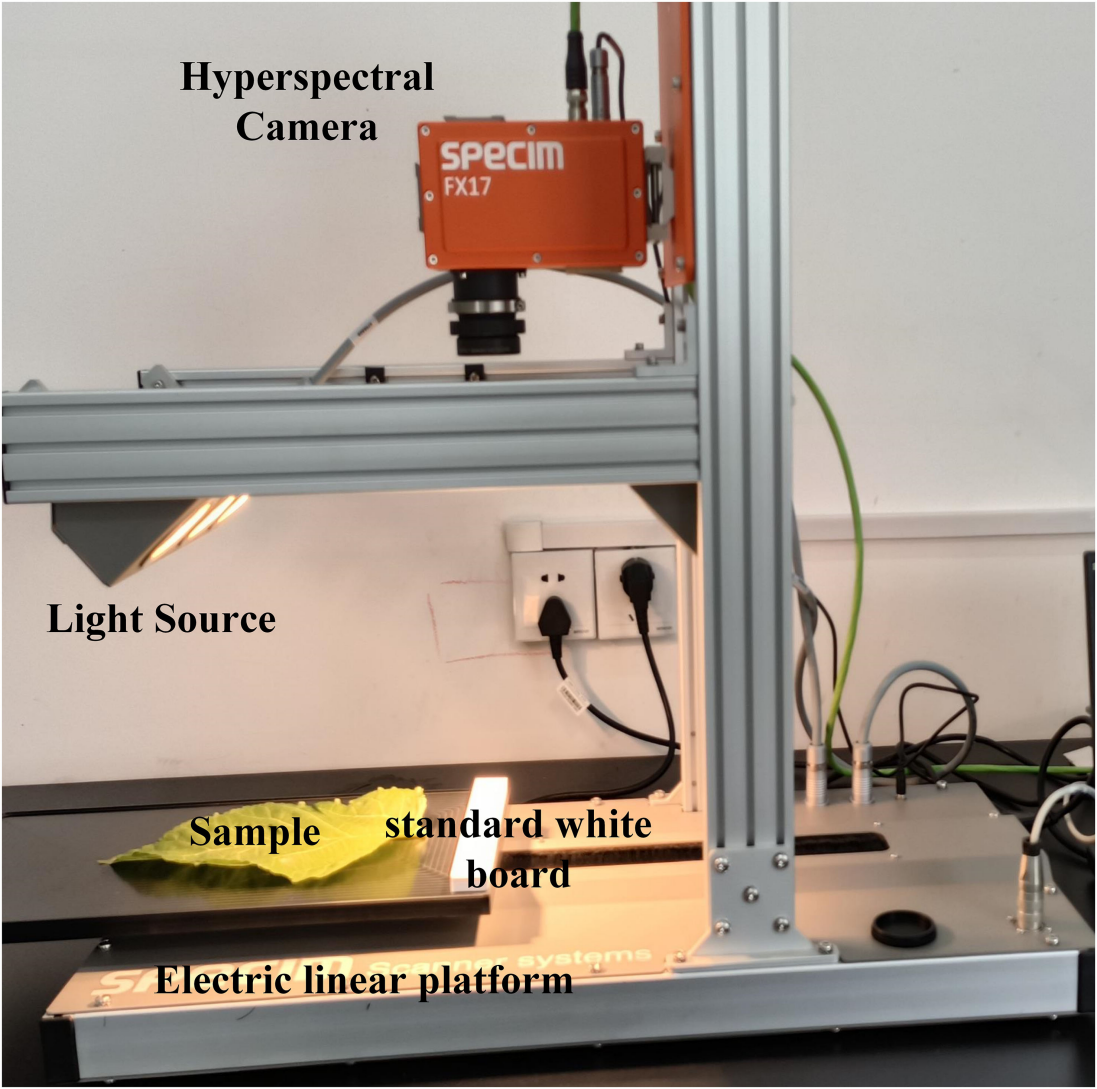
The hyperspectral imaging system.

The HSI acquisition system consists of two hyperspectral cameras (FX10 and FX17, Spectral Imaging Ltd., Oulu, Finland), the electric linear platform (Spectral Imaging Ltd., Oulu, Finland), two light sources (each light source consists of three 20-W halogen lamps), and a laptop (**Figure 1**). The FX10 spectral camera (Si detector) is used for acquiring hyperspectral images of the Vis-NIR region (400–1,000 nm). The FX17 spectral camera (InGaAs detector) is used for acquiring hyperspectral images of the SWIR region (900–1,700 nm).

The two light sources are at an angle of 45° of the moving platform, and the distance between the lens and the platform is 33 cm. When FX10 is used for hyperspectral image acquisition, the exposure time is set to 12.5 ms, the frame rate is 49.83 Hz, the spectral interval is 2, the spatial interval is 1, and the mobile platform moving speed is set to 11.9 mm/s. When FX17 is used for hyperspectral image acquisition, the exposure time is set to 6 ms, the frame rate is 40.5 Hz, the spectral interval is 1, the spatial interval is 1, and the moving speed of the mobile platform is set to 14.8 mm/s. The hyperspectral image acquisition is conducted after the preheating of 20 min. The white reference image W is obtained by screening the standard white board with a reflectance of 99% placed in front of the sample. The dark reference image D is obtained by screening with the lens closed. The reference images are acquired together with the hyperspectral image of the sample.

**Figure 2:**
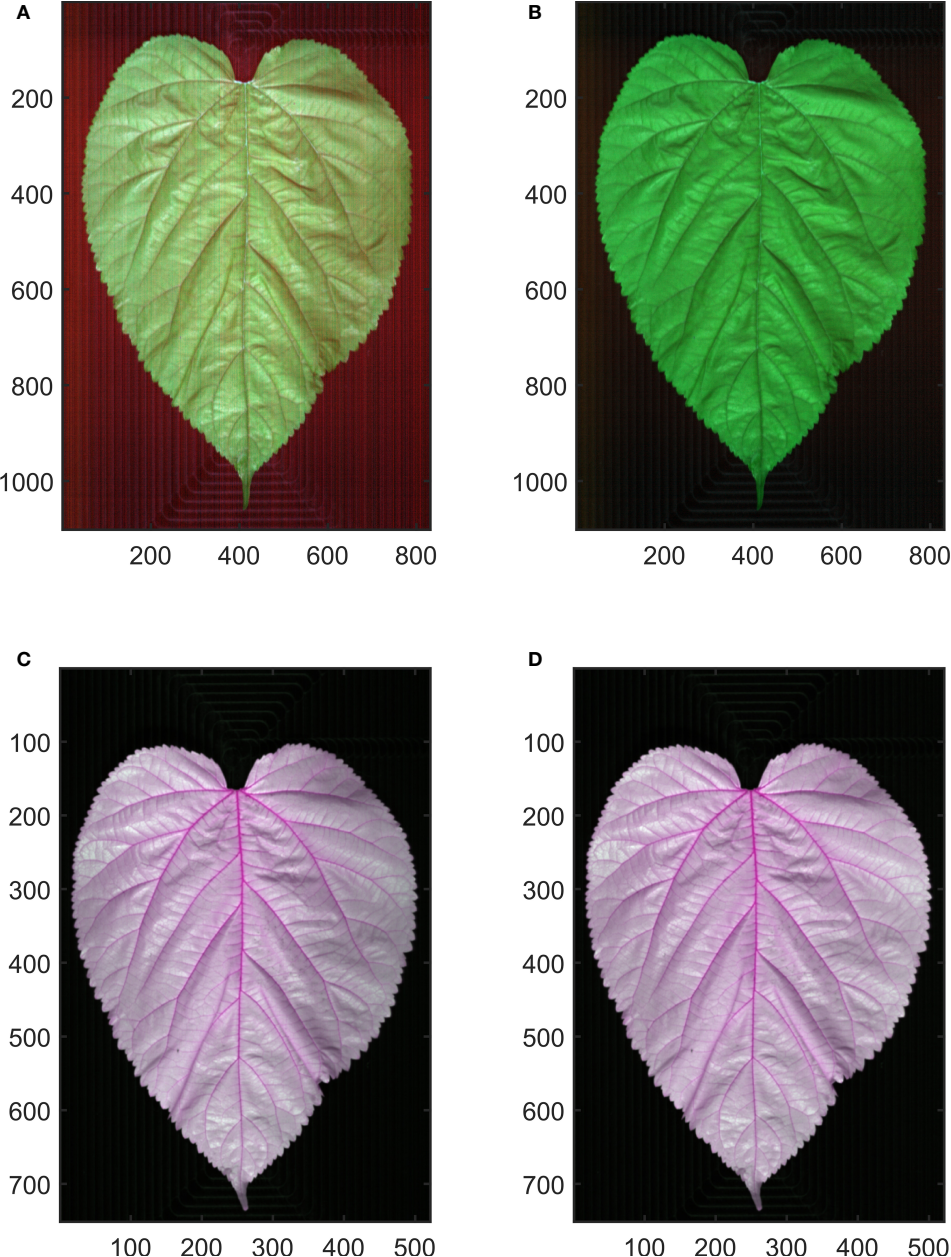
Hyperspectral image calibration. **(A, C)** show the raw hyperspectral image of Vis-NIR and SWIR region, respectively. **(B, D)** show the calibrated hyperspectral image of Vis-NIR and SWIR region, respectively.


To avoid the effect caused by uneven light source intensity distribution and dark current during the image collecting process, hyperspectral image calibration is conducted (**Figure 2**). The following formula is used for hyperspectral image calibration.

$${\text{R}}_{\text{λ}}=\frac{{\text{I}}_{\text{λ}}-{\text{D}}_{\text{λ}}}{{\text{W}}_{\text{λ}}-{\text{D}}_{\text{λ}}}$$

where *R_λ_* is the calibrated image, *I_λ_* is the raw image, *W_λ_* is the white reference image, and *D_λ_* is the dark reference image.

### 2.3 Determination of protein content of mulberry leaves

After the hyperspectral image acquisition, the mulberry leaves underwent drying in the oven at 105°C for 15 min and then drying at 50°C for 2 h. With the main vein removed, the leaves were ground with a mortar and passed through a 60-mesh sieve, and the protein content of mulberry leaves was determined by using Kaye nitrogen determination (Chromý et al., 2015). The sample (0.3 g) was weighed and transferred into a digestion tube. Then, 0.4 g of copper sulfate, 6 g of potassium sulfate, and 20 mL of sulfuric acid were added to the tube for digestion. Once the temperature of the digestion furnace reached 420°C, the digestion process was continued for 1 h. After the liquid in the digestion tube exhibited a green and transparent appearance, the tube was carefully removed from the furnace and allowed to cool. Once cooled, 50 mL of water was added to the tube. In the Kjeldahl nitrogen analyzer, sodium hydroxide solution, hydrochloric acid standard solution, and boric acid solution containing mixed indicators were first added. Finally, the automated Kjeldahl nitrogen analyzer was utilized to automatically perform the processes of sample addition, distillation, titration, and data recording. The protein content in the mulberry leaf can then be calculated using the provided formula.


$$\text{X}=\frac{(\text{V}1-\text{V}2)*\text{C}*0.0140*\text{F}*100}{\text{m}*\text{V}3/100}$$

In the formula, *X* represents the measured protein content, *V*1 represents the volume of consumed hydrochloric acid standard solution, *V*2 represents the volume of blank consumed hydrochloric acid standard solution, *V*3 represents the volume of extracted liquid. *C* = 0.05 mol/L represents the concentration of hydrochloric acid standard solution. *m* represents the weight of the sample taken. *F* represents the conversion factor of nitrogen to protein, and *F* is taken as 6.25. 100 is the conversion factor.

### 2.4 Data processing

#### 2.4.1 Region of interest identification and spectrum extraction

In this study, a whole mulberry leaf is the region of interest for spectral extraction. A gray image is obtained at 800 nm and 1,000 nm of the Vis-NIR and SWIR hyperspectral images, respectively. The Otsu method automatically calculates the segmentation threshold between the leaf and the background in the gray image, from which a binary image is obtained. Then, the ROI is obtained by conducting mask processing. Finally, the average spectral reflectance of the whole mulberry leaf at each wavelength is calculated.

#### 2.4.2 Spectral pretreatment

In light of the high noises in the first and last bands of the original spectral data, spectral data within the ranges of 423–975 nm (Spectral Range I), 970–1,684 nm (Spectral Range II), and 423–1,684 nm (Spectral Range III) are selected for subsequent analyses. The raw spectra need to be pre-treated to eliminate the scattering caused by uneven particle distribution and different particle sizes and the influence of optical path difference on the spectral data. The standard normal variate (SNV) (Barnes et al., 1989), Savitzky–Golay combined first-order derivation (Savitzky and Golay, 1964), and multiplicative scatter correction (MSC) (Isaksson and Næs, 1988) are the commonly used spectral preprocessing methods, and have been shown to be effective in eliminating or reducing interference such as electrical noise, sample background, and stray light during acquisition. In order to determine the best pretreatment of spectral data, the SNV, Savitzky–Golay combined first-order derivation, and MSC are adopted in this study.

#### 2.4.3 Variable selection

Because of the high correlation between adjacent spectral bands, successive projections algorithm (SPA), competitive adaptive reweighted sampling (CARS), and random frog (RF) are respectively used to extract characteristic wavelengths in this study to reduce model input variables and improve model efficiency and prediction accuracy.

SPA is a forward variable selection method, which selects a wavelength at the beginning, calculates the projection value of the remaining wavelength, cycles forward, selects the wavelength corresponding to the maximum projection value, and then combines the projection vector with the wavelength until the end of the cycle (Araújo et al., 2001). SPA can minimize the collinearity between variables, extract the minimum redundant information variable group, and reduce the number of variables required to establish the model, thus improving the efficiency and speed of modeling.

CARS is a feature variable selection method that combines Monte Carlo sampling with PLS model regression coefficient (Li et al., 2009). The primary selection of the feature variables is conducted by combining the PLS regression coefficient with exponential decay through adaptive weighted sampling each time. Then, a new PLS model is constructed based on the new subset established with the points of larger absolute weight of regression coefficient retained and the points with smaller weight removed in the PLS model. After multiple calculations, the wavelength in the subset with the smallest root mean square error of the PLS model is selected as the characteristic wavelength.

RF is a very effective algorithm, which is similar to Reversible Jump Markov Chain Monte Carlo, proposed by Li for variable selection of high-dimensional data (Li et al., 2012). It functions in an iterative manner, calculating the probability of each variable being selected in each iteration. The higher the probability, the greater the importance of the variable. The variable with the higher probability is preferred as the characteristic variable.

#### 2.4.4 Model construction and assessment

In this study, PLS and least squares-support vector machine (LS-SVM) are selected for constructing models. LS-SVM (Suykens and Vandewalle, 1999), a machine learning algorithm based on support vector machine, is selected for constructing a regression model by adopting partial least squares linear system as loss function through nonlinear mapping function. With input variables projected into a high-dimensional feature space, and then the optimization problem converted into equality constraint conditions, this model has good generalization performance and nonlinear regression processing performance. When LS-SVM is used for analysis, appropriate kernel functions must be decided. In this study, RBF kernel function is adopted, and two parameters of the kernel function, γ and σ2, are selected by grid searching based on cross-validation. PLS (Mehmood et al., 2012), a multivariate statistical analysis method on the basis of principal component analysis, reduces the dimension by projecting independent variables and dependent variables into a new low-dimensional space, thus being capable of being used to treat the linear relationship between multiple independent variables and one or more dependent variables in a high-dimensional data set.

The evaluation metrics of the model are determination coefficient (*R* ^2^), root mean square error (RMSE), and relative percent deviation (RPD). *R* ^2^ reflects the stability of the model. The closer *R* ^2^ is to 1, the better the stability of the model is and the higher the degree of fitting is. RMSE is used for testing the predictive power of the model. The smaller the RMSE is, the better the predictive power of the model is. RPD is the ratio of sample standard deviation to RMSE. When RPD is less than 1.4, the model fails to predict the sample. When 1.4 ≤ RPD< 2, the model is considered to be of average effect and can be used for rough assessment of the samples. When RPD ≥ 2, the model is considered to be of excellent predictive power (Khoshnoudi-Nia and Moosavi-Nasab, 2019).

**Figure 3:**
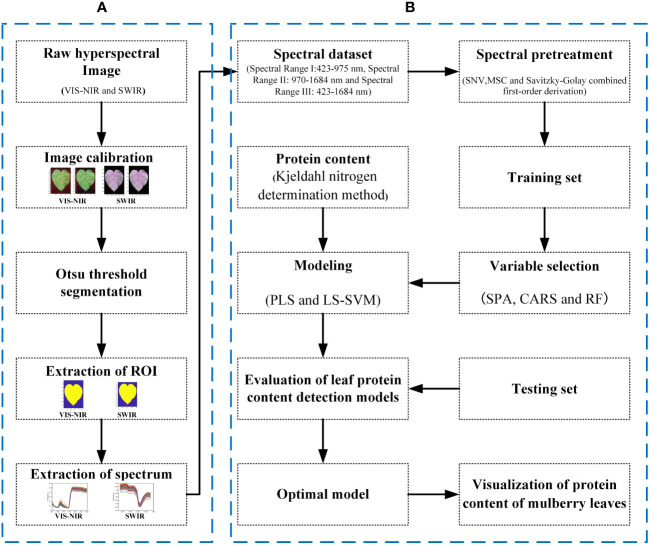
Workflow of data processing. **(A)** The raw hyperspectral image preprocessing and segmentation procedure. **(B)** The spectral processing, variable selection, and the modeling procedure.

The data processing process is shown in **Figure 3**. The calibration of the hyperspectral images and all the data processing are completed on MATLAB 2022a by encoding.

## 3 Results and analyses

### 3.1 Protein and spectral characteristics of mulberry leaves

**Figure 4:**
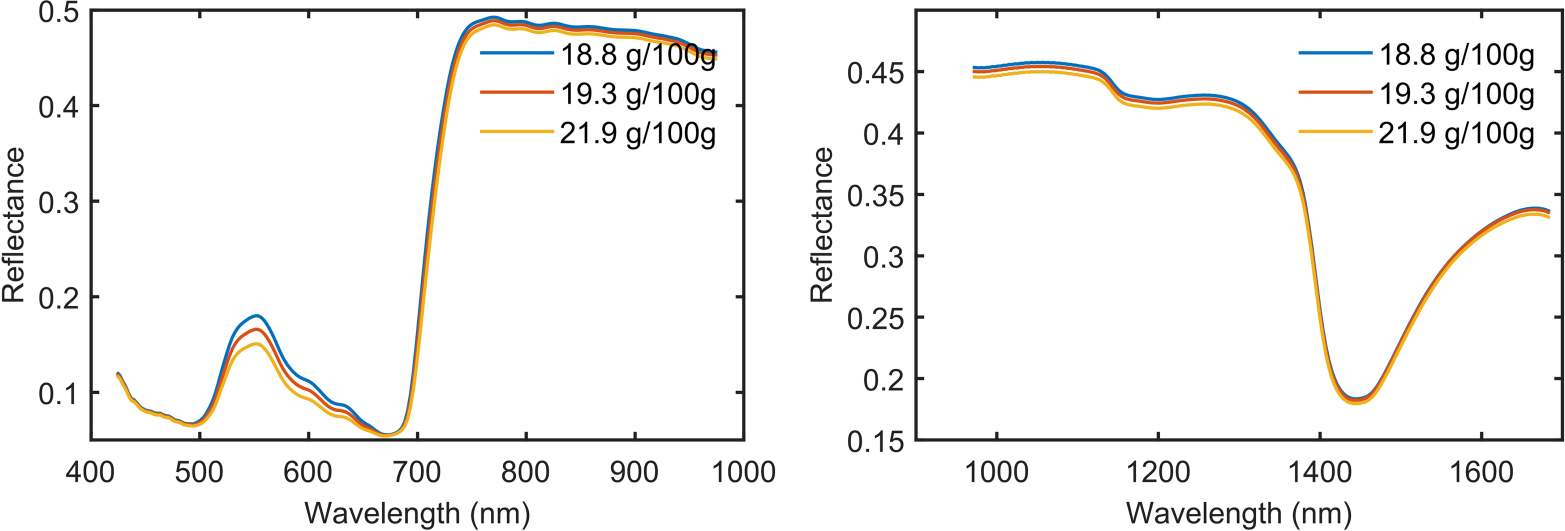
The average spectra of mulberry leaves with different protein content.

The spectral reflection curve is drawn with the samples divided into 3 groups according to the level of protein content (**Figure 4**). The spectral reflection curves of mulberry leaf samples of different protein levels show the characteristics of the typical reflection spectral curve of a green plant, as follows: green peak (530–580 nm), red valley (590–670 nm), red edge (680–780 nm), high reflective platform (750–1,300 nm) related to leaf tissue structures, and the water absorption peak (1,450 nm) (Gates et al., 1965; Gausman and Allen, 1973; Gitelson et al., 1996; Tang et al., 2005). Absorption peaks of protein-associated N-H bonds have been reported at 1,020 nm and 1,510 nm in the SWIR region, but this absorption peak is not directly shown from the spectrum of **Figure 4**, which may be due to the fact that the absorption bands in the NIR region tend to be wide and often overlap (Curran, 1989; ElMasry et al., 2011). In addition, from **Figure 4**, we can see that the higher the protein content of mulberry leaves is, the lower the corresponding spectral reflectance is.

### 3.2 Results of feature wavelength extraction

**Figure 5:**
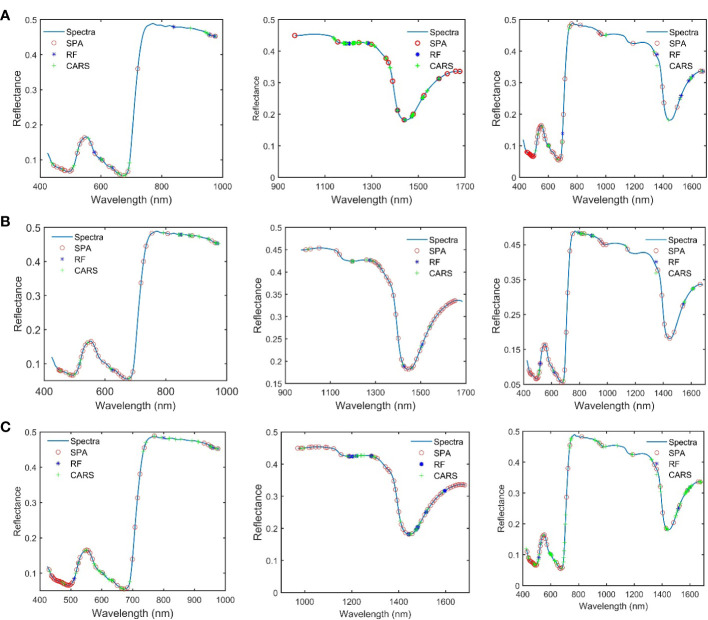
The characteristic wavelength obtained after the combination of different pretreatment and variable extraction methods. **(A)** SNV preprocessing. **(B)** Savitzky–Golay combined first-order derivation preprocessing. **(C)** MSC preprocessing.

In this study, SPA, RF, and CARS are being used individually to extract characteristic wavelengths from spectral data within three band ranges (**Figure 5**). In this study, the subset of bands with the smallest root Mean Square Error of Cross-Validation (RMSECV) value was selected as the characteristic band determined in the CARS and SPA algorithms. The CARS algorithm was iterated 1,000 times to ensure a comprehensive exploration. Similarly, the RF algorithm was also iterated 1,000 times to thoroughly explore the entire dataset, and by selecting the top 10 wavelength variables with a high average probability from these 1,000 runs, we obtained the characteristic wavelengths.

Analysis of the feature wavelengths extracted by using SPA, RF, and CARS shows that there are differences in the positions and numbers of the obtained feature wavelengths extracted from the spectral data undergoing the same pretreatment by using the different feature screening methods, but the extracted wavelength positions tend to concentrate in some specific bands. There are also differences in the positions and numbers of the obtained feature wavelengths extracted from the spectral data undergoing different pretreatments by using the same feature screening method, but the extracted wavelength positions tend to concentrate in some specific bands. The obtained feature wavelengths extracted in Spectral Range I mainly concentrate in 450–700 nm and 900–1,000 nm. The obtained feature wavelengths extracted in Spectral Range II mainly concentrate in 1,170–1,350 nm. The obtained feature wavelengths extracted in Spectral Range III bands mainly concentrate in the visible light region of 450–700 nm, near 800 nm, at 950 nm, and in 1,500– 1,650 nm. It is found, that the characteristic bands of proteins obtained in relevant studies are highly overlapping in the positions with the characteristic wavelengths obtained in this study. However, there are obvious differences in the specific positions and numbers. This is speculated to be caused by the heterogeneity of protein composition among different species (Caporaso et al., 2018; Ma J. et al., 2019; Ma et al., 2021; Cruz-Tirado et al., 2023). These results demonstrate the effectiveness of the applied feature screening methods (SPA, RF, and CARS) in extracting relevant wavelengths for protein content detection in mulberry leaves using HSI.

### 3.3 Results of modeling

Prediction models for protein content is constructed on the basis of PLS and LS-SVM, respectively, by combining three pretreatment methods, three feature wavelength screening methods, and full-band wavelength (**Tables 1**–**3**). In this study, the *R* ^2^, RMSE, and RPD of the test set are used to evaluate the predictive ability of the model, and the most suitable model for mulberry leaf content detection is selected by combining the number of variables and the predictive ability of the model.

**Table 1:** Results of models in spectral range I.

Pretreatment	Variable selection	No. of variables	PLS				LS-SVM		
			PCs	*R* ^2^	RMSE	RPD	*R* ^2^	RMSE	RPD
SNV	Full bands	204	17	0.82	1.14	2.28	0.60	1.71	1.60
	SPA	24	21	0.89	0.91	2.62	0.86	1.01	2.69
	CARS	23	18	0.88	0.93	2.68	0.84	1.09	2.53
	RF	10	9	0.88	0.92	2.81	0.84	1.08	2.49
S.G. first-order derivation	Full bands	204	16	0.90	0.85	2.94	0.70	1.47	1.85
	SPA	35	23	0.90	0.85	2.91	0.82	1.15	2.36
	CARS	26	11	0.66	1.57	1.63	0.69	1.51	1.80
	RF	10	7	0.69	1.49	1.61	0.72	1.43	1.91
MSC	Full bands	204	16	0.86	1.01	2.59	0.60	1.71	1.59
	SPA	51	18	0.90	0.87	2.87	0.76	1.31	2.08
	CARS	39	14	0.87	0.96	1.96	0.73	1.40	1.95
	RF	10	8	0.89	0.90	3.20	0.85	1.04	2.63

**Table 2:** Results of models in spectral range II.

Pretreatment	Variable selection	No. of variables	PLS				LS-SVM		
			PCs	*R* ^2^	RMSE	RPD	*R* ^2^	RMSE	RPD
SNV	Full bands	204	15	0.86	1.02	2.17	0.87	0.97	2.81
	SPA	15	15	0.92	0.78	3.07	0.93	0.71	3.83
	CARS	26	9	0.85	1.05	1.95	0.86	1.03	2.24
	RF	10	8	0.81	1.19	2.15	0.80	1.21	2.65
S.G. first-order derivation	Full bands	204	14	0.85	1.04	2.13	0.89	0.90	3.04
	SPA	42	19	0.86	1.01	2.25	0.91	0.80	3.40
	CARS	13	7	0.85	1.05	2.12	0.88	0.94	2.91
	RF	10	7	0.80	1.20	1.84	0.86	1.01	2.70
MSC	Full bands	204	14	0.88	0.94	2.36	0.87	0.98	2.78
	SPA	45	15	0.92	0.78	3.09	0.86	1.02	2.68
	CARS	26	8	0.81	1.17	1.93	0.81	1.17	2.34
	RF	10	9	0.74	1.37	1.48	0.71	1.44	1.89

**Table 3:** Results of models in spectral range III.

Pretreatment	Variable selection	No. of variables	PLS				LS-SVM		
			PCs	*R* ^2^	RMSE	RPD	*R* ^2^	RMSE	RPD
SNV	Full bands	405	18	0.90	0.84	3.04	0.88	0.93	2.93
	SPA	50	21	0.92	0.78	3.20	0.92	0.78	3.51
	CARS	19	14	0.75	1.36	1.40	0.70	1.48	1.30
	RF	10	10	0.67	1.54	1.78	0.39	2.10	1.84
S.G. first-order derivation	Full bands	204	22	0.93	0.72	3.39	0.91	0.82	3.31
	SPA	43	28	0.92	0.77	3.20	0.90	0.85	3.22
	CARS	19	7	0.87	0.97	2.49	0.85	1.04	2.62
	RF	10	6	0.88	0.95	2.61	0.88	0.92	2.97
MSC	Full bands	204	16	0.90	0.86	3.00	0.87	0.97	2.80
	SPA	48	19	0.93	0.73	3.51	0.86	0.99	2.75
	CARS	77	16	0.92	0.76	3.39	0.87	0.99	2.75
	RF	10	9	0.79	1.24	1.79	0.29	2.28	1.19

It can be seen in **Table 1**, in Spectral Range I, the results obtained using the PLS models are better than those obtained using the LS-SVM models, and the S.G. first-order derivation + SPA + PLS model achieves the best performance, with an *R* ^2^ of 0.90, an RMESP of 0.85, and an RPD of 2.91. It can be seen in **Table 2**, in Spectral Range II, the results obtained using LS-SVM and PLS models are not much different, and the SNV + SPA + LS-SVM model achieves the best performance, with an *R* ^2^ of 0.93, an RMESP of 0.71, and an RPD of 3.83. It can be seen in **Table 3**, in Spectral Range III, the results obtained by using the PLS model are better than those obtained by using the LS-SVM model, and the SNV + SPA +L S-SVM model achieves the best performance, with an *R* ^2^ of 0.93, an RMESP of 0.73, and an RPD of 3.51. The above results show that there is no specific pretreatment method, characteristic wavelength, or modeling method optimal for all types of spectral data, and it is necessary to explore the effects of different algorithm combinations on model performance, so as to select the optimal processing method in light of different situations. LS-SVM is superior to linear methods in solving nonlinear problems, but it is sensitive to noise and error in the input data, while in Spectral Range I and III, the data used for modeling may be of poor quality or poorly correlated with the detection of protein content spectral data, so PLS algorithms obtain better results than LS-SVM algorithms (Suykens et al., 2002; Wang and Hu, 2005).

As can be seen from **Tables 1**–**3**, better results are achieved by using the models based on the feature extraction method, compared with the full-band models, which is due to the fact that the full-band spectral data have some redundant and interference information, and this is an indication that the feature extraction method can effectively reduce the redundant information between adjacent spectral bands and improve the accuracy of models. Two hyperspectral cameras with different wavelength ranges were compared, and the overall performance of the predictive model developed in the SWIR region shows better predictive power and robustness than that established in the Vis-NIR region, which is exactly opposite to the results of Ma et al. (Ma J. et al., 2019). They obtained better results in detecting pork protein by using spectral data of the Vis-NIR region. However, in many other protein detection studies, good prediction results are obtained by using spectral data of the SWIR region (Talens et al., 2013; Ma et al., 2021; He et al., 2023). In this study, compared with the models constructed based on Spectral Range II spectral data, the model based on Spectral Range III spectral data fails to show better accuracy, although it obtains richer spectral information. This may result from the spectrum of the Spectral Range III region containing more redundant information related to the detection of protein content of mulberry leaves. The above results show that the SWIR region is the optimal spectral range for mulberry leaf protein prediction.

Previous studies have explored the feasibility of HSI for the non-destructive detection of protein content; however, few studies have attempted to determine the optimal spectral range for measuring proteins, especially for fresh mulberry leaves. In this study, the best results are obtained by combining the SWIR HSI acquisition system based on InGaAs detectors with SNV + SPA + LS-SVM, with an *R* ^2^ of the test set of up to 0.93, an RMSE of only 0.71 g/100 g, and an RPD of up to 3.83. The results show that the model is qualified for detecting and analyzing the protein content of mulberry leaves.

### 3.4 Visualization of protein content of mulberry leaves

**Figure 6:**
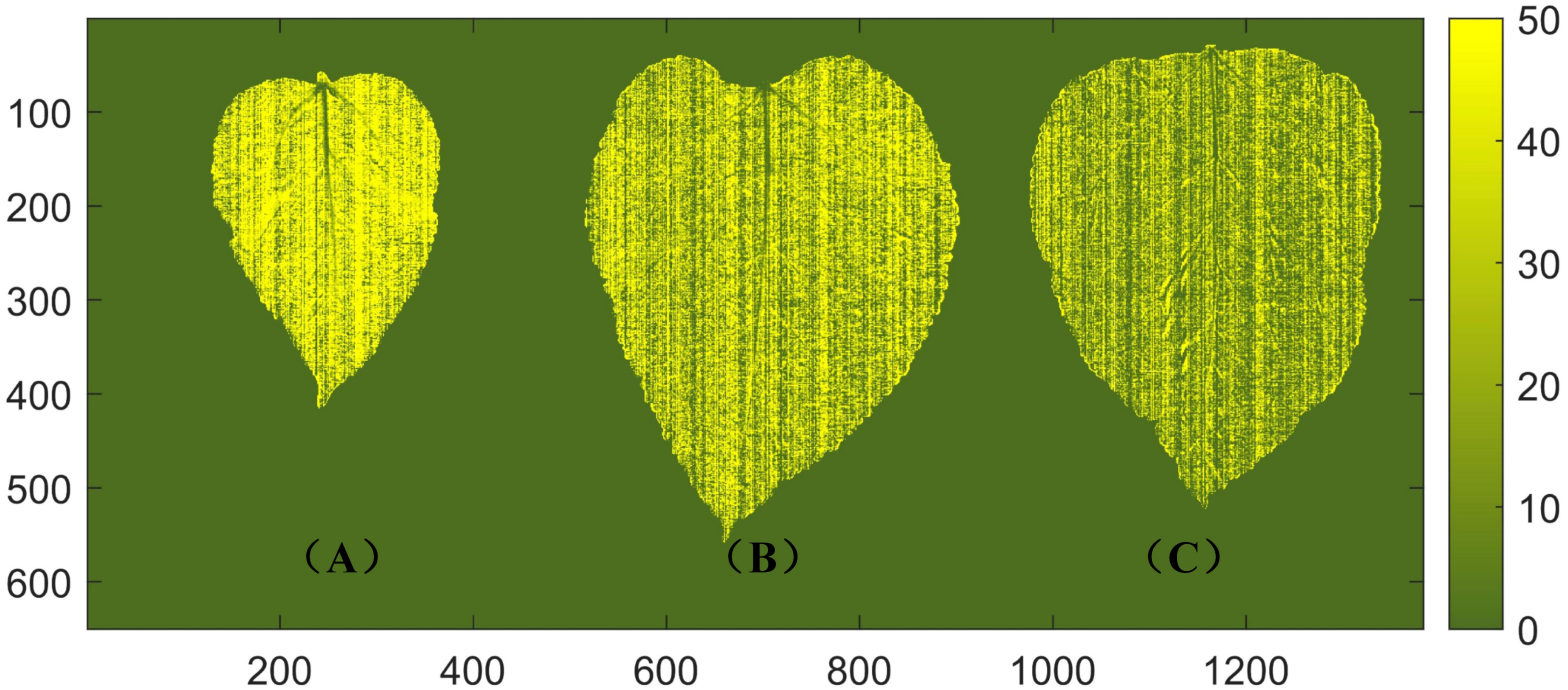
Visualization of protein content in mulberry leaves. **(A)** A young leaf with a protein content of 45.7 g/100 g. **(B)** A middle mature leaf with a protein content of 26.3 g/100 g. **(C)** A mature leaf with a protein content of 16.3 g/100 g.

The distribution of protein content in mulberry leaves has not been reported. In the practical application of non-destructive detection technology for mulberry leaf protein, the visualization of the protein content of mulberry leaves can not only provide valuable insights for merchants to classify the freshness and quality of mulberry leaves more intuitively, but also aid researchers in conducting plant physiology studies related to mulberry leaves. By extracting spectral data from all pixels of the leaves, a distribution map is generated by using the established SNV + SPA + LS-SVM model to visualize the spatial distribution of protein content in mulberry leaves. The level of protein content is represented by the depth of shade, as depicted in **Figure 6**. It should be noted that the variety, harvest time, and maturity significantly influence the nutrient content of mulberry leaves. Previous studies have indicated that the protein content of mulberry leaves decreases with increasing ripeness (Ramesh et al., 2021). In the visualization results of this study, it can be seen that tender leaves exhibit higher protein content compared to mature leaves, which is consistent with the above findings. Additionally, the visualized distribution of the protein content of mulberry leaves shows that the protein of healthy mulberry leaves is essentially evenly distributed in the mesophyll, while the protein content in the veins is extremely low. This is due to heterogeneity, and the fact that the protein content varies across different locations within the sample and the leaf vein is mainly composed of cellulose and conductive substances with no capacity of storing energy (Fukuda, 2004; Jiang et al., 2022).

## 4 Conclusion

The protein content of mulberry leaves is a crucial indicator for assessing their quality. In this study, we aimed to develop a rapid and non-destructive method for detecting the protein content of mulberry leaves using HSI technology. The feasibility of using HSI technology within the spectral range of 400–1,000 nm and 900–1,700 nm for non-destructive detection of mulberry leaf protein content is investigated. By comparing different spectral ranges of the HSI acquisition system and utilizing various data processing methods, including preprocessing, variable extraction, and modeling, prediction models for protein content detection are constructed. The results demonstrated that the best performance was achieved by combining the spectral data from 900–1,700 nm with SNV + SPA + LS-SVM. This approach yielded a testing set *R* ^2^ value of up to 0.93, an RMSE of only 0.71 g/100 g, and an RPD of up to 3.83. Furthermore, the visualization of the protein content distribution in mulberry leaves based on the best model revealed that healthy leaves exhibited an even distribution of protein content throughout the mesophyll, with lower protein concentrations observed in the leaf veins.

These findings show the optimal spectral range for mulberry leaf protein prediction and highlight the potential of utilizing SWIR HSI combined with the SNV–SPA–LS-SVM algorithm for rapid, non-destructive, and high-precision detection of protein content in mulberry leaves. The developed method can provide valuable insights for assessing the quality of mulberry leaves in a non-invasive manner, enabling efficient monitoring and optimization of mulberry leaf quality.

## Data availability statement

The raw data supporting the conclusions of this article will be made available by the authors, without undue reservation.

## Author contributions

XL: Conceptualization, Software, Writing – original draft. FP: Data curation, Investigation, Writing – original draft. ZW: Investigation, Writing – original draft. GH: Conceptualization, Funding acquisition, Supervision, Writing – review & editing. JL: Methodology, Supervision, Writing – review & editing.
